# Supplementary material for: Dynamic analysis and optimal control of stochastic information cross-dissemination and variation model with random parametric perturbations
Source: PLoS One. 2024 May 23;19(5):e0303300. doi: 10.1371/journal.pone.0303300 (PMC11115323; doi:10.1371/journal.pone.0303300)
Supplement: Attachment — Submitted filename: Review Report_PONE-D-23-34170.pdf [file pone.0303300.s002.pdf]

Review Report

Manuscript ID: PONE-D-23-34170

This paper considers that there are many stochastic factors in the social system, which will result in the phenomena of information cross-dissemination and variation. The dual-system stochastic susceptible-infectious-mutant-recovered (2S2I4M2R) model of information cross-dissemination and variation is derived from this problem. Afterward, the existence of the global positive solution is demonstrated, sufficient conditions for the disappearance of information and its stationary distribution are calculated, and the optimal control strategy for the stochastic model is proposed. The numerical simulation supports the results of the theoretical analysis and is compared to the parameter variation of the deterministic model. The results demonstrate that cross-dissemination of information can result in information variation and diffusion. Meanwhile, white noise has a positive effect on information dissemination, which can be improved by adjusting the perturbation parameters.

There is some new contribution and may be consider for publication after addressing the following observations.

1. In abstract the author claims that white noise has a positive effect on information dissemination, which can be improved by adjusting the perturbation parameters. They may provide the evidence for this either theoretically or numerically. In the current version I did not see any evidence for this claim.

2. The introduction should also make a compelling case for why the study is useful along with a clear statement of its novelty or originality by providing relevant information and providing answers to basic questions such as:
   i. What is already known in the literature?
   ii. What was done and how it was done?

3. The author may also add some recants work about the current study related to their work in introduction part. They may add the following but not mandatory.
   https://doi.org/10.1038/s41598-023-41861-4
   https://doi.org/10.1063/1.5016680

https://doi.org/10.1016/j.aej.2023.01.027
doi: 10.3934/math.2023210

4. Even though all the figures are available at the end, but there is no figure in the text and only captions of the figures are there, please see page from 3 onwards.

5. From page 7 onwards there is too many mathematical equations. The author may delete the unnecessary mathematical equations.

6. Improve the quality of the figures (from figure 4 and onwards)

7. In numerical simulation section, what is the criteria to choose the values of the parameters involve in model equation (1) and equation (5).

8. Which parameter is more sensitive and why in numerical simulations section. The author must write some sentences about this.

9. Author may look for some punctuation, typos and editing issues.

10. The conclusion section is too long. In general conclusion consist of what is claimed is achieved.
