# Supplementary material for: Dynamic analysis and optimal control of stochastic information cross-dissemination and variation model with random parametric perturbations
Source: PLoS One. 2024 May 23;19(5):e0303300. doi: 10.1371/journal.pone.0303300 (PMC11115323; doi:10.1371/journal.pone.0303300)
Supplement: Attachment — Submitted filename: Response to Reviewers.pdf [file pone.0303300.s003.pdf]

Response to Reviewers

Dear Editors and Reviewers:

Thank you very much for your letter and the reviewers' comments concerning our manuscript entitled " Dynamic Analysis and Optimal Control of Stochastic Information Cross-dissemination and Variation Model with Random Parametric Perturbations" (ID: PONE-D-23-34170). These comments are indeed extremely helpful for revising and improving our paper, as well as the important guiding significance to our researches. We have studied these comments with care and have made correction which we hope meet with approval. The main corrections in the paper and the responds to the reviewer's comments are as flowing:

Responds to the reviewer's comments:

Reviewer #1:

Response to comment: (The reviewer appreciates the paper's presentation of experiments designed by author which seems to contributing the valuable insights to stability inference in a stochastic world model. The reviewer also commends the paper for contributing to the understanding of information dynamics in social systems.)

Response: Thank you very much for your recognition of our manuscript. We will continue to explore the application of random perturbations in real-world social systems. In addition, we will also conduct more in-depth research in the field of information dissemination. Thank you again for your encouragement.

Special thanks to you for your good comments!

Reviewer #2:

1. Response to comment: (Language requires extensive revision. There are many typos and repeated words as in the paragraph between lines 16 and 26. Other examples are shown in the attached PDF file.)

Response: Thanks very much for your comments. Your comments are conducive us to be more careful. It is more helpful for us to improve our good scientific research literacy. According to your request, we have revised upon all typos and repeated words once again. And we have already put them right in the revised version. As these minor errors are more, we have not marked each and every of them corresponding page and line numbers in details.

2. Response to comment: (Reference 30 cited in the literature review but there is no clear relation with the current study.)

Response: Thank you for helping us to find the errors. We are very sorry for not being careful to make this obvious mistake. Reference 30 really does not have a clear relationship with the research in this article. According to your suggestions and requirements, **we have deleted reference 30.**

3. Response to comment: (Figures 4 to 9 are displayed in a very low resolution and hence many information are not clear.)

Response: Thank you very much for your careful reading, helpful comments and constructive suggestions, which has significantly improved the presentation of our manuscript. In the original manuscript, our image resolution was indeed very low, resulting in the information displayed being not clear enough. For this, we are very sorry for the inconvenience caused to your reading. According to your comments and suggestions, **we have further improved the resolution of all the figures and inserted them into the revised manuscript (Figure 1 to 10).**

Special thanks to you for your good comments!

Reviewer #3:

1. Response to comment: (In abstract the author claims that white noise has a positive effect on information dissemination, which can be improved by adjusting the perturbation parameters. They may provide the evidence for this either theoretically or numerically. In the current version I did not see any evidence for this claim.)

Response: Thank you very much for your comments! Your suggestions are of great help to the improvement of the paper. In the original manuscript, our expression is not comprehensive and careful enough. We would like to make a further explanation on this point. According to your request, **we have further explained how white noise effect on information dissemination in numerical simulations in the revised manuscript (Page 20, line 413 - 420).** The details are as follows:

"In Figure 5 and Figure 7, the fluctuating lines represent the population density changes after adding random disturbances. And the stable lines represent the population density changes without adding random disturbances. It can be seen that the population densities with added random perturbations are higher than that without added random perturbations. From Figures 5 (c)-(d) and Figure 7 (c)-(d), the white noise disturbance enhances the population densities of information dissemination group I_1 and I_2. From this, it can be seen that white noise disturbance has a positive effect on information dissemination."

2. Response to comment: (The introduction should also make a compelling case for why the study is useful along with a clear statement of its novelty or originality by providing relevant information and providing answers to basic questions such as:

i. What is already known in the literature?

ii. What was done and how it was done?)

Response: Thank you very much for your incisive comments and thorough reminders. Your comments are extremely important for improving the practical application of the model established in this paper. According to your comments and suggestions, **we have added a clear statement of its novelty or originality in the revised manuscript (Page 3, line108 - Page 4, line 124).** The details are as follows:

"In social systems multiple pieces of information sometimes coexist. The

phenomenon of coexistence and intersection in the process of multiple information dissemination is similar to the spread of viruses in biology. For example, when the SARS-CoV-2 virus spread in 2020, the presence of its original strain did not cause the extinction of other strains in the body. Instead, there were multiple strains coexisting, and even new mutated strains were produced through cross transmission, such as Omicron (B.1.1.529). The phenomenon of coexisting and producing mutated strains of this virus can be analogized and applied to the spread of multiple information. Multiple similar information may have coexisting relationships, and after prolonged cross propagation, the content expressed by each information may deviate from the original information, similar to the phenomenon of multiple viruses coexisting and producing mutated viruses. This paper proposes a stochastic susceptible-infectious-mutant-recovered model that considers information cross-dissemination and variation, and then demonstrates the existence of global positive solutions. After calculating the sufficient conditions for information disappearance and information stationary distribution, the appropriate parameters are selected as control variables. The numerical simulation validates the rationality of the proposed method is finally validated through numerical simulation."

3. Response to comment: (The author may also add some recants work about the current study related to their work in introduction part. They may add the following but not mandatory.

https://doi.org/10.1038/s41598-023-41861-4

https://doi.org/10.1063/1.5016680

https://doi.org/10.1016/j.aej.2023.01.027

doi: 10.3934/math.2023210)

Response: Thanks for your comments and suggestions. We have downloaded and read these references. These references are closely related to the research of this paper, and they deepen our understanding. It is more helpful for us to clearly understand the the application of stochastic differential equations. **Therefore, we have added refers to these useful recent papers in the revised manuscript (Page 2, line 60 - 69).** The details are as follows:

“Chu et al.(2023)[30] constructed an $S_f M_b M_g U$ malnutrition model with random perturbations and crossover effects, and using fractional differential equations analysis deterministic-stochastic model. Rashid et al.(2023)[31] constructed an $SI_p I_q I_{qp} R_p R_q R_{qp} B$ of the co-infection of the fractional pneumonia and typhoid fever disease stochastic model with cost-effective techniques and crossover effects. Ali et al.(2023)[32] gave the dynamics analysis and simulations of stochastic COVID-19 epidemic model using Legendre spectral collocation method. In addition, Khan et al.(2018)[33] studied on the application of Legendre spectral-collocation method to delay differential and stochastic delay differential equation.”

4. Response to comment: (Even though all the figures are available at the end, but there is no figure in the text and only captions of the figures are there, please see page from 3 onwards.)

Response: Thank you very much for your careful reading, helpful comments and constructive suggestions, which has significantly improved the presentation of our manuscript. In the original manuscript, we did not put the figures in the text. For this, we are very sorry for the inconvenience caused to your reading. According to your comments and suggestions, **we have inserted all the figures into the main text in the revised manuscript (Figure 1 to 10).**

5. Response to comment: (From page 7 onwards there is too many mathematical equations. The author may delete the unnecessary mathematical equations.)

Response: Thank you very much for the attentive and earnest comments! Your suggestions will help improve the reading experience of the manuscript. Because we want to fully reflect the work of this manuscript as much as possible in the article. Therefore, the necessary steps were included in the main text. Your reminder can help us learn simpler steps to prove in future research. We will continue to study the papers you have provided us and delve into the proof methods from these excellent papers.

6. Response to comment: (Improve the quality of the figures (from figure 4 and onwards).)

Response: Thank you very much for your incisive comments and thorough reminders. We are very sorry that our image resolution was indeed very low, resulting in the

information displayed being not clear enough. According to your comments and suggestions, **we have further improved the resolution of all the figures in the revised manuscript (Figure 1 to 10).**

7. Response to comment: (In numerical simulation section, what is the criteria to choose the values of the parameters involve in model equation (1) and equation (5).)

Response: Thank you very much for the attentive and earnest comments! We do regret for the lack of smooth logical narration due to the omission of necessary instructions in the original manuscript, which has brought you a dissatisfactory reading experience. According to your comments and suggestions, **we have added instructions for the criteria to choose the values of the parameters involve in model equation (1) and equation (5) in the revised manuscript (Page 18, line 381 - Page 19, line 384).** The details are as follows:

"Due to the range of the parameters has not been explicitly given in previous studies. Therefore, the values of the parameters in the model can be given according to the conditions given by Theorem 3.1 and Theorem 4.1."

8. Response to comment: (Which parameter is more sensitive and why in numerical simulations section. The author must write some sentences about this.)

Response: Thanks very much for your comments. We do regret for the lack of explanation of parameters sensitivity due to the omission of necessary instructions in the original manuscript, which has brought you a dissatisfactory reading experience. According to your comments and suggestions, **we have added some sentences to explanation of parameters sensitivity in the revised manuscript (Page 20, line 421 - 430).** The details are as follows:

"Next, in order to verify the effectiveness of the optimal control strategy, and then observe the change of densities of $I_1(t)$ and $I_2(t)$ when the optimal control strategy is adopted. Here, the optimal control is adopted for the control variables $\alpha_{11}, \alpha_{12}, \alpha_{22}, \alpha_{21}$ and other parameters remain unchanged. Figure 9 and Figure 10 confirm the densities of $I_1(t)$ and $I_2(t)$ change over time when $\sigma_i(i = 1\sim8) = 0.001$ and $\sigma_i(i = 1\sim8) = 0.0001$ under constant control measure and optimal control. From Figure 9 and Figure 10, it can be seen that adopting optimal control for

control parameter $\alpha_{11}, \alpha_{12}, \alpha_{22}, \alpha_{21}$ can further enhance information dissemination. That is to say, the cross-contact rate is more sensitive to the dissemination of information."

9. Response to comment: (Author may look for some punctuation, typos and editing issues.)

Response: Thanks very much for your comments. Your comments are conducive us to be more careful. It is more helpful for us to improve our good scientific research literacy. According to your request, we have revised upon all punctuation, typos and editing issues once again. And we have already put them right in the revised version. As these minor errors are more, we have not marked each and every of them corresponding page and line numbers in details.

10. Response to comment: (The conclusion section is too long. In general conclusion consist of what is claimed is achieved.)

Response: Thanks for your sincere comments and reminders, which are indeed to the point. We agree that the conclusion of this article is indeed too long. According to your comments and suggestions, **we have reduced the textual description in the conclusion section and highlighted the key points more in the revised manuscript (Page 21, line 442 - 460).** The details as following:

"This study investigates the influence of multi-population information cross-dissemination, mutation, and white noise disturbance on information dissemination. And developed the stochastic model. The following results are achieved through examination of this paper: (1) White noise disturbance can facilitate the information dissemination, and stochastic environmental factors play a positive role in information dissemination. (2) As disturbance intensity increases, the stochasticity of the model gradually enhances, and the fluctuation of information dissemination trend becomes more apparent. (3) The information dissemination may be effectively facilitated by controlling the perturbation parameters; unlike earlier research, the optimal control strategy provided in this paper is based on the optimal value calculated by the control variables.

To sum up, for positive information, it is necessary to give full play to the activity of the social system itself, and to introduce a large number of stochastic components into the social system to improve the information dissemination. For negative information, on the other hand, it is crucial to eliminate the external environment's uncertain factors and reduce the impact of uncontrollable factors on the social system in order to inhibit its dissemination. In future study, the role of stochastic environmental factors on information dissemination in social systems will be further investigated, and a $L\grave{e}vy$ process-driven information dissemination model will be developed.”

Special thanks to you for your good comments!

Reviewer #4:

1. Response to comment: (The abbreviation for the suggested model is unclear, and I believe it should be omitted from the text.)

Response: Thanks very much for your comments. We do regret for the unclearly explanation of abbreviation for the suggested model in the original manuscript, which has brought you a dissatisfactory reading experience. We deeply apologize for this. According to your comments, **we have omitted the abbreviation for the suggested model from the text in the revised manuscript.** As these minor errors are more, we have not marked each and every of them corresponding page and line numbers in details.

2. Response to comment: (The introduction lacks consistency and quality in its writing; thus, it requires significant improvement.)

Response: Thanks very much for your comments. Your comments are conducive us to be more careful. It is more helpful for us to improve our good scientific research literacy. We do regret for the lack of consistency due to the omission of necessary instructions in the original manuscript, which has brought you a dissatisfactory reading experience. According to your comments and suggestions, **we have made significant improvement to the introduction and added necessary explanations in the revised manuscript (Page 2, line 60 - 69).** The details are as follows:

"In social systems multiple pieces of information sometimes coexist. The phenomenon of coexistence and intersection in the process of multiple information dissemination is similar to the spread of viruses in biology. For example, when the SARS-CoV-2 virus spread in 2020, the presence of its original strain did not cause the extinction of other strains in the body. Instead, there were multiple strains coexisting, and even new mutated strains were produced through cross transmission, such as Omicron (B.1.1.529). The phenomenon of coexisting and producing mutated strains of this virus can be analogized and applied to the spread of multiple information. Multiple similar information may have coexisting relationships, and after prolonged cross propagation, the content expressed by each information may deviate from the original information, similar to the phenomenon of multiple viruses coexisting and producing mutated viruses. This paper proposes a stochastic susceptible-infectious-mutant-

recovered model that considers information cross-dissemination and variation, and then demonstrates the existence of global positive solutions. After calculating the sufficient conditions for information disappearance and information stationary distribution, the appropriate parameters are selected as control variables. The numerical simulation validates the rationality of the proposed method is finally validated through numerical simulation."

3. Response to comment: (The construction of the Lyapunov function in Theorem 1 requires revision.)

Response: Thank you very much for the attentive and earnest comments! Your comments further deepened our understanding of the construction of the Lyapunov function. According to your comments and suggestions, we further studied the method of the construction of the Lyapunov function. By our understanding， there is no unified standard and rule for constructing Lyapunov function. According to the conditions to be satisfied by Lyapunov function, we have made a lot of attempts. The method we choose just satisfy the conditions of Lyapunov function. We choosed a simple and effective way to construct Lyapunov function in this paper. We will try our best to study the construction of Lyapunov function by other methods in the future. **We have reconstructed the Lyapunov function in Theorem 1 in the revised manuscript (Page 7, line 215 - Page 8, line 239).**

4. Response to comment: (The threshold parameter determining the existence of a stationary solution should be based on stochastic perturbations.)

Response: Thanks for your sincere comments and reminders, which are indeed to the point. We have indeed overlooked the impact of random perturbations on the threshold of information stationary distribution. According to your comments, **we have re-proved Theorem 4.1. The necessary and sufficient conditions for the stationary distribution of information are described through random perturbation parameters in the revised manuscript (Page 11, line 285 - Page 14, Line 323).**

Special thanks to you for your good comments!

We tried our best to improve the manuscript and made some changes in the manuscript. These changes will not influence the content and framework of the paper.

We appreciate for Editors/Reviewers' warm work earnestly, and hope that the correction will meet with approval.

Once again, thank you very much for your comments and suggestions.
